# A novel circular RNA (hsa_circ_0059930)-mediated miRNA–mRNA axis in the lipopolysaccharide-induced acute lung injury model of MRC-5 cells

**DOI:** 10.1080/21655979.2021.1916276

**Published:** 2021-05-18

**Authors:** Jianhua Han, Shigeng Li, Yawei Feng, Yufeng He, Weilong Hong, Zhiqiang Ye

**Affiliations:** a Department of Emergency, The Third Affiliated Hospital, Sun Yat-sen University, Guangzhou, China; b Department of Anesthesiology, The Third Affiliated Hospital, Sun Yat-sen University, Guangzhou, China

**Keywords:** Circular RNA, hsa_circ_0059930, lipopolysaccharide, acute lung injury, topoisomerase 1, RNA sequencing

## Abstract

Circular RNA (circRNA) is a class of newly discovered endogenous non-coding RNA with closed circular structure. Some circRNAs have been reported to be closely associated with acute lung injury (ALI). While the expression profile of circRNAs in lipopolysaccharide (LPS)-induced ALI and the underlying roles are still not completely clear. The LPS-induced ALI model of MRC-5 cells was first established, and the expression profiles of circRNAs and mRNAs in LPS-induced MRC-5 cells were confirmed through RNA sequencing analysis. Gene Ontologyanalysis and Kyoto Encyclopedia of Genes and Genomes pathway analysis were also applied to predict the latent functions and pathways of the differential mRNAs. After hsa_circ_0059930 knockdown, the proliferation and apoptosis of MRC-5 cells were identified by CCK-8, flow cytometer, and western blot assays. And we predicted the network analysis of hsa_circ_0059930. We testified that LPS could markedly prevent proliferation and induce apoptosis of MRC-5 cells. We discovered a total of 820 differential circRNAs (560 upregulated and 260 downregulated circRNAs) and 484 differential mRNAs (240 upregulated and 244 downregulated mRNAs) in LPS-induced MRC-5 cells. Besides, hsa_circ_0059930 was identified to be significantly upregulated in LPS-induced MRC-5 cells, and knockdown of hsa_circ-0059930 could notably accelerate proliferation and suppress apoptosis of LPS-mediated MRC-5 cells. Moreover, through the network analysis of hsa_circ_0059930, we preliminarily screened the potential regulatory axis hsa_circ_0059930/hsa-miR-382-5p/Topoisomerase 1 (TOP1) in LPS-induced ALI. Our data contribute to understand the importance of circRNAs and mRNAs in LPS-induced ALI. We also provided many hsa_circ_0059930-mediated microRNA (miRNA)–mRNA axis, especially hsa_circ_0059930/hsa-miR-382-5p/TOP1 in LPS-induced ALI.

## Graphical abstract

**Figure:**
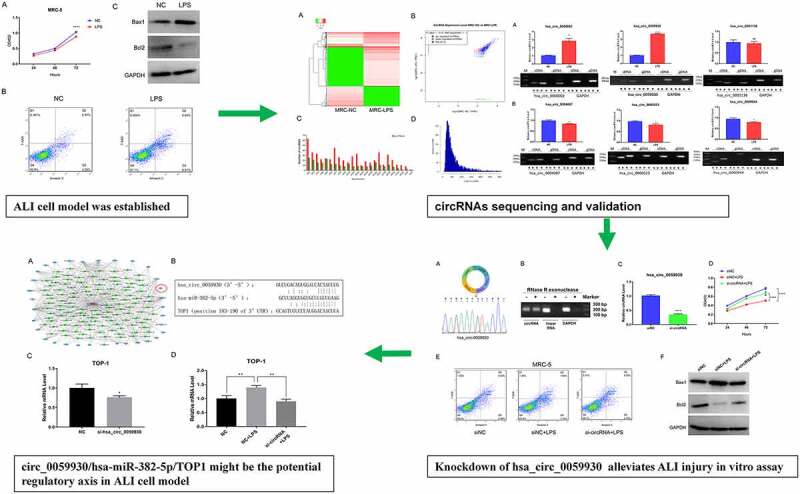

## Introduction

Sepsis is a systemic inflammatory response syndrome (SIRS), which is caused by severe trauma, shock, massive blood loss, reperfusion injury, and major surgical operation. Lung is the most vulnerable target organ in sepsis. And acute lung injury/acute respiratory distress syndrome (ALI/ARDS) is an early stage of sepsis. ALI is acute and progressive respiratory failure caused by various pathogenic factors, such as severe infection, shock, acute pancreatitis, trauma, burns, and inhalation of harmful gases [1,2]. ALI is also a comprehensive pulmonary inflammatory response characterized by the increased alveolar-capillary membrane permeability [3]. Clinically, the frequent manifestations of ALI include accelerated respiratory rate, respiratory distress, and refractory hypoxemia [4]. And ARDS is the severe stage of ALI [5]. Currently, the mortality rate of ALI/ARDS has decreased with the improvement of the pathogenesis research and clinical therapeutics, while ALI/ARDS is still one of the main causes of death in critically ill patients [6] The mortality rate of ARDS has been achieved in 50–70% in China [7], also as high as 35–40% in developed countries [8]. Researches also indicated that the incidence of ALI/ARDS is 18–38% in septic shock, and the detection rate of bacterial endotoxin [lipopolysaccharide (LPS)] is as high as 51% [9,10]. Therefore, bacterial infection and endotoxin-induced lung injury are the vital pathogenic factors of ALI/ARDS. However, the molecular mechanism of LPS-induced lung injury is not fully understood.

A large number of data exhibited that genes that encode function only account for about 1% of the total genomes, and the remaining transcriptional products are non-coding RNAs (ncRNAs) that do not encode proteins [11,12]. In recent years, ncRNA has become a research hotspot in the field of molecular biology [13] Circular RNA (circRNA), as a special type of ncRNAs, has a closed circular structure [14]. Most circRNAs are composed of exon sequences and are highly conserved in different species [15,16]. Meanwhile, circRNA was specifically expressed in different tissues and developmental stages of different species [17]. Besides, circRNAs, as competing endogenous RNAs (ceRNAs), can competitively bind with microRNAs (miRNAs), thereby regulating the expression of the target genes [18,19]. Currently, vast circRNAs have been proved to play an essential role in the processes of multiple diseases, such as cancer [20], cardiovascular disease [21,22], atherosclerosis [23], alzheimer’s disease [24], etc. Moreover, circRNAs can also participate in the regulation of multiple biological functions, including cell proliferation, metastasis, invasion, apoptosis, inflammation, and drug resistance [25,26]. Another research also reported that hsa_circ_0001434, a novel circRNA, has a significant reduction effect on the inflammatory response in ALI model by targeting has-miR-625-5p to release Wnt/β-catenin and NF-κB [24]. However, plenty of circRNAs have not been well verified in ALI, especially in ARDS. Therefore, many circRNAs in ALI still need to be further explored, their mechanisms and related functions also need to be further verified.

In this study, we further filtrated abundant differential circRNAs and mRNAs using RNA sequencing technology in LPS-induced MRC-5 cells. Potential functions and pathways of the dysregulated mRNAs also were predicted by Gene Ontology (GO) and Kyoto Encyclopedia of Genes and Genomes (KEGG) pathway analysis. Besides, we confirmed the impacts of the selected hsa_circ_0059930 on LPS-induced MRC-5 cells. More importantly, we also predicted the miRNAs–mRNAs, which might be regulated by hsa_circ_0059930 in LPS-induced ALI. Therefore, our results might provide many potential circRNAs and hsa_circ_0059930-miRNA–mRNA axis in LPS-induced ALI, which might be potential biomarkers and therapeutic targets for LPS-induced ALI patients.

## Materials and methods

### Cell culture and transfection

MRC-5 cells were from American Type Culture Collection (ATCC) (CCL-171, Manassas, VA). And MRC-5 cells were cultured in Eagle’s Minimum Essential Medium (ATCC, cat. no. 30-2003) containing 10% fetal bovine serum (FBS, Hyclone, cat. no. SH30071.03) and 1% penicillin streptomycin solution (Invitrogen, cat. no. 15,140-122) in an incubator with 5% CO_2_ at 37°C. Cell models of lung injury were constructed in the cultured MRC-5 cells, which were administrated with 100 ng/ml LPS (Sigma, St. Louis, MO, cat. no. L2880) at 37°C for 24 h. MRC-5 cells in negative control (NC) group were added with phosphate belanced solution (PBS). A corresponding siRNA negative control (siNC) (5ʹ-UUCUCCGAACGUGUCAGGUUU-3ʹ) and the siRNA targeting hsa_circ_0059930 (5ʹ-GUUCAGAAAGUACCAUGCAUU-3ʹ) were synthesized from RiboBio Co., Ltd. (Guangzhou, China) for knockdown of circ_0008934 expression. Cells were seeded into a 6-well plate until approximately 60% confluence, and then transfected with NC or hsa_circ_0059930 siRNAs by using Lipofectamine 3000 (Invitrogen).

### Cell counting kit-8 (CCK-8) assay

MRC-5 cells were collected and prepared into a single-cell suspension. MRC-5 cells (5 × 10^4^ cells/mL) were seeded into a 96-well plate and added with or without 100 ng/ml LPS at 37°C for 24 h. After 24, 48, and 72 h of incubation, cells in each well were added with 10 μL CCK-8 solution (Dojindo, Kumamoto, Japan). After an additional 2 h of incubation, we adopted a Microplate Reader (Bio-Rad, Hercules, CA) to measure the absorbance at 450 nm.

### Flow cytometer

MRC-5 cells in NC and LPS groups were harvested and the cell density was adjusted to 1 × 10^9^ cells/L. The MRC-5 cells were then added with 5 μL 7-AAD (7-aminoactinomycin D) and 5 μL Annexin V-APC (Absin, cat no. abs50008) for 10 min away from light. The number of apoptotic MRC-5 cells was identified using the FACS Calibur™ flow cytometer (Becton-Dickinson).

### Western blot

The total protein was extracted from MRC-5 cells in NC and LPS groups using radio-immunoprecipitation assay (RIPA) buffer (Cell Signaling Technology, catno. 9806) containing 1% protease inhibitor (Sigma-Aldrich, cat. no. P8340). Protein concentration was determined by applying the bicinchoninic acid (BCA) protein kit (Sigma, USA, cat. no. Bca1-1kt). Also, 50 μg proteins were detached on the sodium dodecyl sulfate polyacrylamide gel electrophoresis (SDS-PAGE) by electrophoresis (120 V for 90 min), then transferred to the polyvinylidene fluoride (PVDF) membranes. After sealing with 5% skim milk at room temperature for 90 min, the membrane with target protein were added with anti-Bax1 (1:1000; proteintech, cat. no. 50,599-2-lg), anti-Bcl2 (1:2000; Abcam, ab196495), and anti-GAPDH (1:8000; proteintech, cat. no. 60,004-1-lg) overnight at 4°C, respectively. After washing, the membranes were treated with second antibody at room temperature for 60 min. The results were visualized by applying the electrochemiluminescence (ECL) kit (Pierce, cat. no. NCI 5080).

### RNA extraction and purification

Total RNAs were isolated using TRIzol reagent (Invitrogen, CA, USA). The quality of the RNAs was assessed, and the RNA concentration was determined using Nanodrop ND-1000 (NanoDrop Technologies, Wilmington, DE, USA). The purity and integrity of RNA were verified by the 1% denaturing agarose gel electrophoresis. When absorbance ratio of 260/230 was greater than 2.0, absorbance ratio of 260/280 was in the range between 1.8–2.0, and 5S, 16S, and 28S were observed in agarose gel electrophoresis without smear, and RNA was used for further analysis.

### RNA sequencing

The purified RNAs were then handled with the RNase, amplified, and labeled based on the Arraystar’s Super RNA Labeling (Arraystar Inc.). The library was constructed using the purified RNAs, and Qubit® 2.0 fluorometer (Life Technologies, USA) and Agilent 2100 (Agilent Technologies, Palo Alto, CA, USA) were applied to confirm the concentrations and sizes of libraries. Next, the Illumina HiSeq 2000 System was adopted to quantitatively examine the library, and edgeR software was utilized to analyze the differential genes and circRNAs.

### GO and KEGG analysis

GO enrichment of the differential mRNAs was determined through the Bioinformatics Tool (DAVID (Database for Annotation, Visualization and Integrated Discovery), version 6.8, https://david.ncifcrf.gov/) [27,28]. KEGG pathway of the differential mRNAs was confirmed using the KOBAS 2.0 software (http://kobas.cbi.pku.edu.cn/) [29]. The number of enriched differential mRNAs and *P* value was used as the screening criteria.

### RNase R treatment

RNase R (3 U/μg) was added into total RNA (2 μg) and incubated for 10 min at 37°C. Then RNA was used for RT-PCR followed with separation of products in 1.8% agarose gel electrophoresis.

### Reverse transcription polymerase chain reaction (RT-PCR) and uantitative RT-PCR (qRT-PCR)

The purified RNAs were utilized to reverse transcribe into cDNA (complementary DNA) in line with the experimental instructions of the First Strand cDNA Synthesis Kit (TaKaRa, Tokyo, Japan). RT-PCR assay was conducted on the 1.8% agarose gel by electrophoresis, and the results were displayed using the ultraviolet gel imaging system (WEALTEC, Nevada, USA). And the amplification products by divergent primers were applied to conduct Sanger sequencing (TSINGKE, Beijing, China). qRT-PCR assay was performed through SYBR Green qPCR Super Mix (Invitrogen, CA, USA), and the results were gained using the ABI PRISM® 7500 Sequence Detection System. All primer sequences are exhibited in Table 1.

**Table 1.** Primers used for qRT-PCR and RT-PCR methods

Primer ID	Primer sequences (5'–3')
hsa_circ_0000002-CF	AAAGTGGATGAGGAAAGGTGGA
hsa_circ_0000002-CR	GGCGCGGAAGTGTGTCTTG
hsa_circ_0000002-LF	GGAGCTGGAGAGCTACATGG
hsa_circ_0000002-LR	ACTCGCTGTACTTGAGCACC
hsa_circ_0000944-CF	CGGCAGGAGTGGGAGGA
hsa_circ_0000944-CR	CAGCCCAGGCAGCTCG
hsa_circ_0000944-LF	TCCGGGACAGGAACCCTC
hsa_circ_0000944-LR	CTCAATCTCCTGGTAGCGCC
hsa_circ_0001136-CF	AGCCTTTTCACGCTCAAGGT
hsa_circ_0001136-CR	TGAAACCCTCATGTTAAGCAACAA
hsa_circ_0001136-LF	GGCCGAATCAGCCTTTTCAC
hsa_circ_0001136-LR	CAGACCACTCCCAAGCTTACA
hsa_circ_0004087-CF	GAGAACGGGCTCGGTTGAAA
hsa_circ_0004087-CR	TCACAGTGCAAGAGGTGGTG
hsa_circ_0004087-LF	GGCTTCTGGGGACCTTTACG
hsa_circ_0004087-LR	TCACAGTGCAAGAGGTGGTG
hsa_circ_0000523-CF	GTGAGTTCAATGGCTGAGTGC
hsa_circ_0000523-CR	CATCTCCTGCCCAGTCTGACC
hsa_circ_0000523-LF	CCATGTCCCTTGGGAAGGTC
hsa_circ_0000523-LR	TGAGTCTGTTCCAAGCTCCG
hsa_circ_0059930-CF	ACTACATGTTCAGAAAGTACCATGC
hsa_circ_0059930-CR	GTGAGGCTACCCATTGAACCA
hsa_circ_0059930-LF	TCTGCAGACATGCAGGGAAG
hsa_circ_0059930-LR	GAGAGGGAGAACACAAGCCC
GAPDH-CF	CTGAGAACGGGAAGCTTGTC
GAPDH-CR	ACGACCAAATCCGTTGACTC
GAPDH-F	GAGTCAACGGATTTGGTCGT
GAPDH-R	GACAAGCTTCCCGTTCTCAG
TOP1-F	GAGCTGAGCCAGTTGTCCTA
TOP1-R	TTTGCCTGGTAGAACGCTGA

### Identification of miRNA–target interactions

Based on previous research [23,30], we adopted miRanda (version 3.3a) to predict the miRNA–mRNA and miRNA–circRNA interactions in accordance with the conserved target sites, sequence complementarity, and free energy of formation.

### Statistical analysis

The data were emerged as mean ± standard deviation (SD). The results were counted through SPSS 23.0 software (SPSS Inc, Chicago, IL) with Student’s *t*-test or one-way analysis of variance (ANOVA). *P* < 0.05 signified significant difference.

## Results

CircRNAs have been reported to participate in ALI progress. However, many circRNAs in ALI still need to be further explored. In this study, we further filtrated abundant differential circRNAs and mRNAs using RNA sequencing technology in LPS-induced MRC-5 cells. Potential functions and pathways of the dysregulated mRNAs were predicted by GO and KEGG pathway analysis. Then, the impacts of the selected hsa_circ_0059930 on LPS-induced MRC-5 cells were tested by by CCK-8, flow cytometer, and western blot assays. In addition, hsa_circ_0059930–miRNA–mRNA network was constructed, and potential mRNA was further verified. Therefore, our results might provide many potential circRNAs and hsa_circ_0059930–miRNA–mRNA axis in LPS-induced ALI, which might be potential biomarkers and therapeutic targets for LPS-induced ALI patients.

### LPS markedly suppressed proliferation and accelerate apoptosis in MRC-5 cells

**Figure 1.**
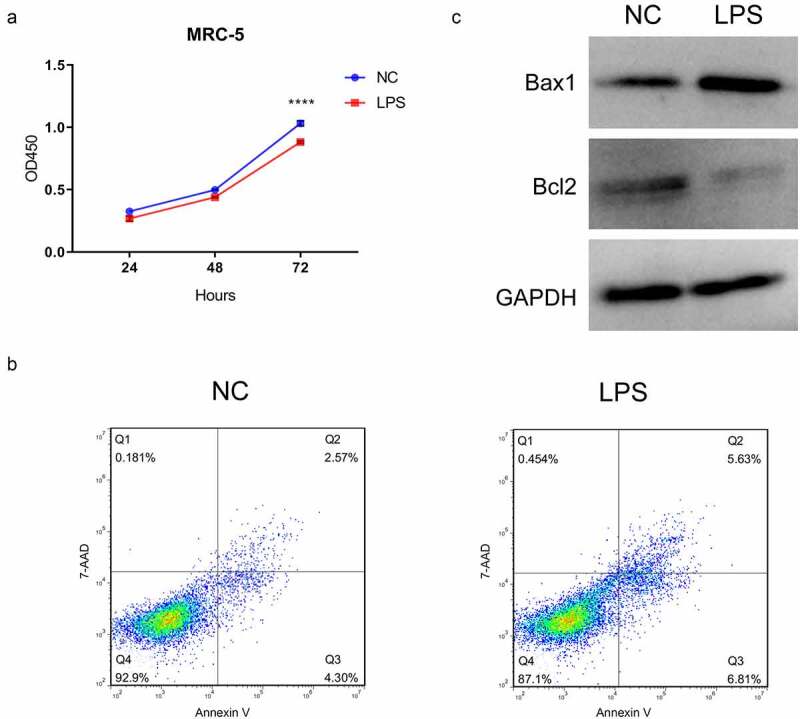
LPS markedly suppressed proliferation and accelerate apoptosis in MRC-5 cells. (a) Proliferation reduction was confirmed by CCK-8 assay in MRC-5 cells when handled with 100 ng/mL LPS for 24 h, ****P < 0.0001. (b) After processing with LPS, the apoptosis of MRC-5 cells was identified through the application of flow cytometer. (c) Western blot analysis revealed the expressions of Bax1 and Bcl2 in LPS-induced MRC-5 cells

In previous study, cell model of ALI can be established through LPS disposition [31]. We also adopted 100 ng/mL LPS to induce MRC-5 cells for 24 h. The data of CCK-8 assay manifested that LPS induction prominently prevented the proliferation of MRC-5 cells (*P* < 0.001, Figure 1a). Also, the data from flow cytometry exhibited that the number of apoptotic MRC-5 cells was markedly increased in the LPS disposition group versus the NC group (Figure 1b). Accordingly, we disclosed that LPS treatment resulted in significant upregulation of Bax1 and significant downregulation of Bcl2 in MRC-5 cells (Figure 1c). Hence, we demonstrated that MRC-5 cell model of ALI has been established by applying LPS.

### Differential expression profile of circRNAs in the LPS-induced and non-induced MRC-5 cells

**Figure 2.**
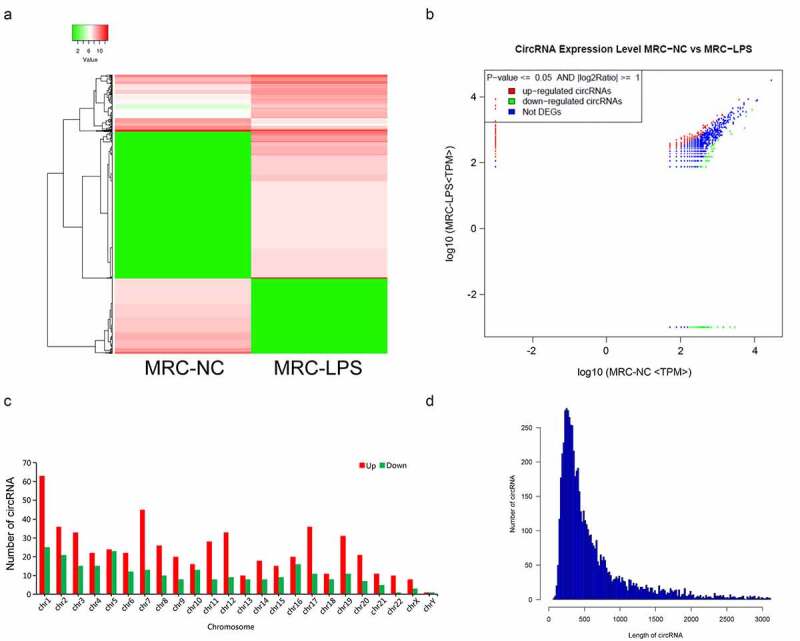
Differential expression profile of circRNAs in LPS-induced and non-induced MRC-5 cells. (a) The expressions of circRNAs in LPS-induced and non-induced MRC-5 cells were presented using the hierarchical clustering analysis in red (high expression) and green (low expression). (b) Volcano plot of differential circRNAs in LPS-induced and non-induced MRC-5 cells. (c) Histogram exhibited the number of differential circRNAs on each chromosome. (d) The length distribution of differential circRNAs in LPS-induced and non-induced MRC-5 cells

To further search the potential circRNAs, the expression profile of circRNAs was created through the RNA sequencing between LPS-induced and non-induced MRC-5 cells. A total of 820 circRNAs were discovered to be differentially expressed in the LPS-induced MRC-5 cells, including 560 upregulated and 260 downregulated circRNAs. And the expression profile of differential circRNAs was also presented through the hierarchical clustering analysis (Figure 2a) and volcano plot (Figure 2b). Meanwhile, we also showed the number of differential circRNAs on each chromosome. And we discovered that most upregulated circRNAs are concentrated in chromosome (chr) 1, chr2, chr3, chr7, chr12, chr17, and chr19, most downregulated circRNAs are concentrated in chr1, chr2, and chr5 (Figure 2c). Simultaneously, the length of most differentially expressed circRNAs was distributed around 500 bp (Figure 2d). Overall, 820 differential circRNAs were screened in the LPS-induced MRC-5 cells, most of which were distributed on chr 1 or chr2, and the length was about 500 bp.

### Identification of six circRNAs with the greatest expression difference in LPS-induced MRC-5 cells

**Figure 3.**
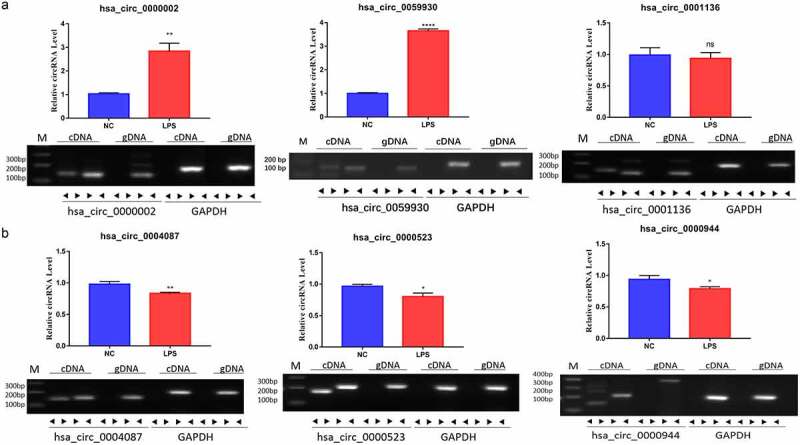
Identification of six circRNAs with the greatest expression difference in LPS-induced MRC-5 cells. (a) qRT-PCR and RT-PCR assays verified the existences and expressions of the three upregulated circRNAs (hsa_circ_0000002, hsa_circ_0059930, and hsa_circ_0001136) in LPS-induced MRC-5 cells. (b) The three downregulated circRNAs (hsa_circ_0004087, hsa_circ_0000523, and hsa_circ_0000944) were also tested by qRT-PCR and RT-PCR assays in LPS-induced MRC-5 cells. *P < 0.05, **P < 0.01, ****P < 0.0001, ns: no significant

In accordance with the expression profile of differential circRNAs in LPS-induced MRC-5 cells, we filtered and validated the top three upregulated circRNAs with the largest difference between two groups (hsa_circ_0000002, hsa_circ_0059930, and hsa_circ_0001136) and the top three downregulated circRNAs with the largest difference between two groups (hsa_circ_0004087, hsa_circ_0000523 and hsa_circ_0000944) using qRT-PCR and RT-PCR assays. Our results uncovered that hsa_circ_0000002 and hsa_circ_0059930 were highly expressed in the LPS model group with respect to the NC group, which conformed to the sequencing results. Meanwhile, the circular structures of hsa_circ_0000002, hsa_circ_0059930, and hsa_circ_0001136 were validated through PCR assay, which hsa_circ_0000002, hsa_circ_0059930, and hsa_circ_0001136 could only be amplified by divergent primers in cDNA (*P* < 0.01, *P* < 0.001, Figure 3a). In addition, our data signified that hsa_circ_0004087, hsa_circ_0000523, and hsa_circ_0000944 were lowly expressed in the LPS model group versus the NC group, which also accorded with the sequencing results. Similarly, we also discovered that hsa_circ_0004087, hsa_circ_0000523, and hsa_circ_0000944 were also only amplified by divergent primers in cDNA, suggesting the existence of circular structure (*P* < 0.05, *P* < 0.01, Figure 3b). Moreover, we discovered that the expression difference of hsa_circ_0059930 was the most obvious according to the verification results.

### Knockdown of hsa_circ_0059930 observably enhanced proliferation and inhibited apoptosis in LPS-induced MRC-5 cells

**Figure 4.**
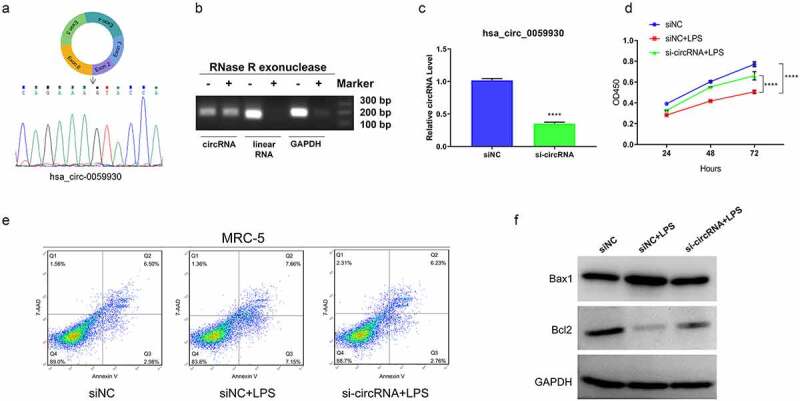
Knockdown of hsa_circ_0059930 observably enhanced proliferation and inhibited apoptosis in LPS-induced MRC-5 cells. (a) According to the formation of hsa_circ_0059930, the back splice structure of hsa_circ_0059930 was confirmed via Sanger sequencing. (b) Stability of hsa_circ_0059930 was confirmed via RNase R treatment assay. (c) After hsa_circ_0059930 interference, the interference effect of hsa_circ_0059930 was determined by qRT-PCR analysis in MRC-5 cells, ****P < 0.0001. (d) CCK-8 assay was applied to confirm the impact of hsa_circ_0059930 silencing on the proliferation of LPS-induced MRC-5 cells, ****P < 0.0001. (e) After hsa_circ_0059930 knockdown, cell apoptosis was determined by applying flow cytometer in LPS-induced MRC-5 cells. (f) The expression changes of Bax1 and Bcl2 were monitored by western blot assay. ****P < 0.0001

Subsequently, we further probed the impacts of hsa_circ_0059930 on the proliferation and apoptosis of LPS-induced MRC-5 cells. Firstly, based on the circbase database (http://www.circbase.org/), we showed the possible formation of hsa_circ_0059930, which was formed from the exons 2–6 on chromosome 20. Next, Sanger sequencing was conducted using the amplification products by divergent primers, and the results proved that hsa_circ_0059930 was circular (Figure 4a). We also confirmed the hsa_circ_0059930 stability via RNase R treatment experiment (Figure 4b). Secondly, hsa_circ_0059930 was silenced in MRC-5 cells through the transfection of hsa_circ_0059930 siRNAs, and the qRT-PCR results also displayed that hsa_circ_0059930 expression was signally reduced in the hsa_circ_0059930 siRNAs group in comparison to the siNC group (*P* < 0.001, Figure 4c). Next, we also certified that knockdown of hsa_circ_0059930 memorably improved the proliferation of LPS-induced MRC-5 cells (*P* < 0.001, Figure 4d). Meanwhile, through the examination of flow cytometer, we testified that knockdown of hsa_circ_0059930 observably weakened the apoptosis of LPS-induced MRC-5 cells (Figure 4e). And the data from western blot also proved that silence of hsa_circ_0059930 dramatically elevated the Bcl2 expression and reduced Bax1 expression in LPS-induced MRC-5 cells (Figure 4f). Overall, we disclosed that knockdown of hsa_circ_0059930 could prominently alleviate LPS-induced ALI in MRC-5 cells.

### The expression profile and function annotation of distinct mRNAs in LPS induced and non-induced MRC-5 cells

**Figure 5.**
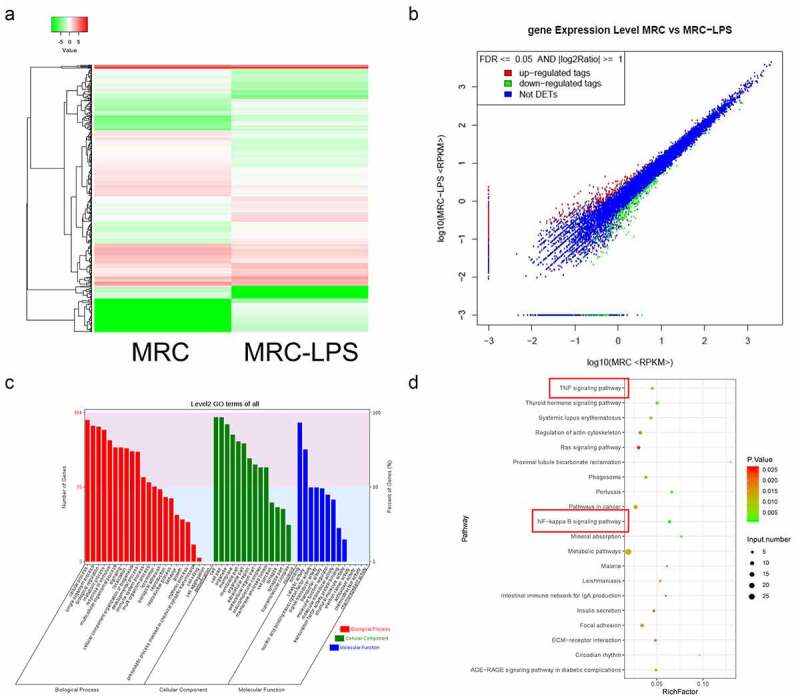
The expression profile and function annotation of distinct mRNAs in LPS-induced and non-induced MRC-5 cells. (a) Based on sequencing results, hierarchical clustering analysis showed the differential mRNAs between MRC-5-NC and MRC-5-LPS groups. (b) Scatterplots of the differential mRNAs in LPS-induced and non-induced MRC-5 cells. Red, green, and blue dots indicate the upregulated, downregulated, and unchanged mRNA, respectively. (c) The GO analysis of the differential mRNAs. (d) Top 20 KEGG pathways were enriched by the differential mRNAs

To further determine the possible downstream molecular mechanisms of hsa_circ_0059930, we further determined the differentially expressed mRNAs between LPS induced and LPS non-induced MRC-5 cells through RNA-sequencing. And compared with MRC-5 cells, a total of 484 differentially expressed mRNAs were discovered in the LPS-induced MRC-5 cells, which contained 240 upregulated mRNAs and 244 downregulated mRNAs. Meanwhile, a hierarchical clustering analysis was utilized to visualize the upregulated or downregulated mRNAs in the LPS model group compared to the NC group (Figure 5a). Also, we applied the scatterplots to show the expression distribution of all mRNAs based on the RNA sequencing data (Figure 5b). Besides, GO analysis was conducted to analyze the underlying roles of differentially expressed mRNAs in the LPS-induced MRC-5 cells. And the GO analysis indicated that the differential mRNAs in LPS-induced MRC-5 cells were mainly associated with multiple biological processes (cellular process, single-organism process, biological regulation, metabolic process, response to stimulus, and multicellular organismal process, etc). And the cellular component of the differential mRNAs mainly enriched in cell, cell part, organelle, membrane, membrane part, and organelle part, etc. In molecular function, the differential mRNAs were mainly relevant to binding, catalytic activity and nucleic acid binding transcription factor activity, etc. (Figure 5c). KEGG pathway analysis also exhibited that the dysregulated mRNAs were mainly related to the signaling pathways, which mainly contained TNF signaling pathway, NF-κB signaling pathway, metabolic pathways, and Ras signaling pathway, etc. (Figure 5d). Therefore, we further screened and annotated the differential mRNAs in the LPS-induced MRC-5 cells.

### hsa_circ_0059930/hsa-miR-382-5p/TOP1 might be the potential regulatory axis in LPS-induced ALI

**Figure 6.**
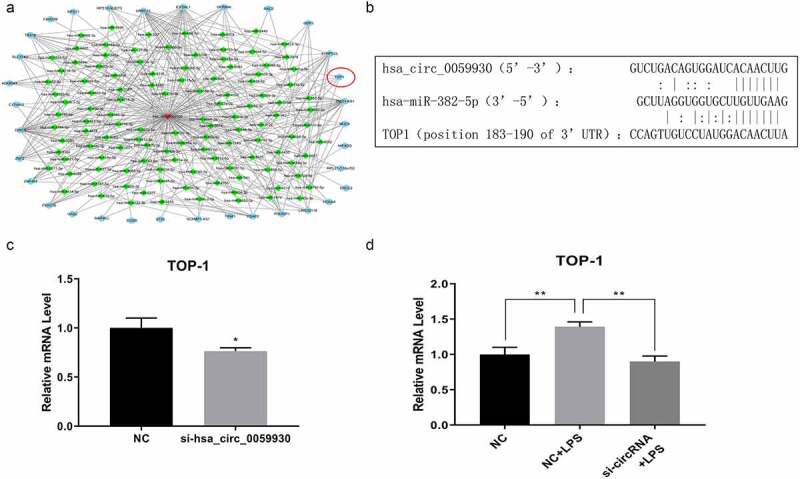
hsa_circ_0059930/hsa-miR-382-5p/TOP1 might be the potential regulatory axis in LPS-induced ALI. (a) Based on the circRNA and mRNA sequencing results, the related network of hsa_circ_0059930 was analyzed. (b) Through bioinformatics analysis, we predicted and displayed the potential binding sites between hsa-miR-382-5p and hsa_circ_0059930 or between hsa-miR-382-5p and TOP1. (c) qRT-PCR assay was employed for the silence identification of hsa_circ_0059930 in MRC-5 cells, *P < 0.05. (d) qRT-PCR analysis was also applied to assess the influence of hsa_circ_0059930 interference on the TOP1 expression in LPS-induced MRC-5 cells, **P < 0.01

Combined with circRNA and mRNA sequencing data, we further conducted bioinformatics analysis of possible target genes of hsa_circ_0059930. And the network analysis of hsa_circ_0059930 is displayed in Figure 6a and Supplementary Table 1. It has been reported that TOP1 can significantly regulate TNF and NF-κB [32,33]. Subsequently, TOP1 was screened through a comprehensive analysis of KEGG pathways. Also, we predicted that hsa-miR-382-5p had common binding sites with hsa_circ_0059930 and TOP1 through bioinformation analysis. In accordance with the network analysis, we preliminarily filtrated the hsa_circ_0059930/hsa-miR-382-5p/TOP1 regulation axis in LPS-induced MRC-5 cells. Additionally, we presented the potential binding sites between hsa-miR-382-5p and hsa_circ_0059930 or between hsa-miR-382-5p and TOP1 (Figure 6b). Next, we also proved that knockdown of hsa_circ_0059930 could significantly downregulate TOP1 in MRC-5 cells, which was in line with the expected results of RNA sequencing (*P* < 0.05, Figure 6c). Above all, we confirmed that the level of TOP1 was dramatically elevated in LPS group relative to the NC group, while the elevation of TOP1 in LPS-induced MRC-5 cells also could be prominently attenuated by hsa_circ_0059930 knockdown (*P* < 0.01, Figure 6d). Hence, we revealed that the hsa_circ_0059930/hsa-miR-382-5p/TOP1 axis might be associated with the ALI.

## Discussion

Sepsis is the leading cause of death in critically ill patients. And ALI/ARDS is the most frequent complication of sepsis [34]. In clinical practice, no drugs with definite therapeutic effect have been discovered, and no standard treatment regimen has been established [35]. Therefore, it is urgent to explore the mechanism of ALI in sepsis. In our study, we adopted MRC-5 cells to establish the lung injury model through the induction of LPS based on the previous study [31]. We also testified that LPS could markedly inhibit proliferation and facilitate apoptosis of MRC-5 cells.

CircRNA, as a class of ncRNA with a closed structure, has been reported to be relevant to plentiful disease processes, including cardiovascular disease [21], neurological disease [15], and cancer [20]. With the development of sequencing technology, a growing number of circRNAs have been discovered [15,36]. In our study, we further adopted RNA sequencing to analyze the expressions of circRNAs and mRNAs in LPS-induced and non-induced MRC-5 cells. A total of 560 upregulated and 260 downregulated circRNAs were identified, 240 mRNAs were upregulated, and 244 mRNAs were downregulated in LPS-induced MRC-5 cells. After the verification of qRT-PCR and RT-PCR assays, we found that hsa_circ_0000002 and hsa_circ_0059930 were upregulated, hsa_circ_0004087, hsa_circ_0000523, and hsa_circ_0000944 were downregulated in LPS-induced MRC-5 cells. hsa_circ_0059930 was also selected as the research target based on the degree of expression difference. We also verified that hsa_circ_0059930 silencing could notably enhance proliferation and suppress apoptosis of LPS-induced MRC-5 cells. Therefore, hsa_circ_0059930 might have a potential therapeutic effect for LPS-induced ALI.

CircRNA has also been proven to have a variety of biological functions, such as regulating transcription or splicing, acting as a sponge for miRNA, interacting with RNA-binding proteins and translating proteins [37]. In our study, we also conducted the network analysis of hsa_circ_0059930 and predicted a total of 261 potential hsa_circ_0059930–miRNA–mRNA axis in LPS-induced ALI. Moreover, based on the GO and KEGG analysis of the differential mRNAs, we suggested that the dysregulated mRNAs mainly were related to the cell process and participated in the TNF and NF-κB signaling pathways. In the target genes, TOP1, as an enzyme in the nucleus, can catalyze the breakage and reconnection of DNA strands, and change the topological structure of DNA, and the superhelical structure of chromosomes [38]. It was reported that TOP1 has a significant effect in multiple biological processes, such as gene duplication, transcription, chromosome separation, and DNA repair [39,40]. Researches showed that topotecan, as a TOP1 inhibitor, can alleviate LPS-induced ALI by regulating NF-κB signaling pathway [33]; the activity of TOP1 can be inhibited by chemotherapeutic drugs to suppress the genes, which are regulated to cytokine and NF-κB [32]. Therefore, we speculated that TOP1 expression is in connection with ALI, which can be induced by hsa_circ_0059930. Through the bioinformatics analysis and qRT-PCR validation, we preliminarily determined that hsa_circ_0059930/hsa-miR-382-5p/TOP1 might be the underlying regulatory mechanism in LPS-induced ALI.

However, our study only preliminarily screened many potential differential circRNAs, mRNAs, and hsa_circ_0059930-related miRNAs/mRNAs, and preliminarily determined hsa_circ_0059930/hsa-miR-382-5p/TOP1 axis in LPS-induced MRC-5 cells. The relationship between hsa_circ_0059930 and hsa-miR-382-5p, hsa-miR-382-5p, and TOP1 should be verified via double luciferase activity assay and RNA IP experiment. The specific functions and mechanisms of hsa_circ_0059930/hsa-miR-382-5p/TOP1 axis also need to be further validated through *in vivo* and *in vitro* experiments.

## Conclusion

In conclusion, we for the first time defined and screened out the differential circRNAs and mRNAs in LPS-induced MRC-5 cells and revealed that the dysregulated mRNAs mainly were relevant to the TNF and NF-κB signaling pathways and participated in cell process. Meanwhile, we suggested that the identified hsa_circ_0059930 might be the novel therapeutic targets for the LPS-induced ALI. Moreover, we predicted 261 potential hsa_circ_0059930-related miRNA/mRNA axis, especially hsa_circ_0059930/hsa-miR-382-5p/TOP1 axis in LPS-induced MRC-5 cells. Therefore, our research may provide potential circRNAs and possible regulatory axes for the diagnosis and treatment of ALI.

## Supplementary Material

Supplemental Material

## Data Availability

The data sets generated during and/or analyzed during the current study are available from the corresponding author on reasonable request. And the circRNAs and mRNAs profile data were deposited at NCBI under BioProject number PRJNA726358.

## Highlights

1. LPS-treated MRC-5 cells can be the cell model of ALI.
2. circ_0059930 was significantly upregulated in LPS-treated MRC-5 cells.
3. Silence of circ-0059930 promotes proliferation but inhibits apoptosis in ALI.
4. circ_0059930/miR-382-5p/TOP1 might be potential regulatory axis in ALI.

## Abbreviations

LPS: lipopolysaccharide
circRNA: Circular RNA
ALI: acute lung injury
GO: gene ontology
KEGG: kyoto encyclopedia of genes and genomes
CCK-8: cell counting kit-8
TOP1: topoisomerase 1
SIRS: systemic inflammatory response syndrome
ALI/ARDS: acute lung injury/acute respiratory distress syndrome
ncRNAs: non-coding RNAs
ceRNAs: competing endogenous RNAs
miRNAs: microRNAs
FBS: fetal bovine serum
NC: negative control
siNC: siRNA negative control
PBS: phosphate belanced solution
7-AAD: 7-Aminoactinomycin D
RIPA: radio-immunoprecipitation assay
BCA: bicinchoninic acid
SDS-PAGE: sodium dodecyl sulfate polyacrylamide gel electrophoresis
PVDF: polyvinylidene fluoride
ECL: electrochemiluminescence
DAVID: Database for Annotation, Visualization and Integrated Discovery
RT-PCR: reverse transcription polymerase chain reaction
qRT-PCR: quantitative RT-PCR
cDNA: complementary DNA
SD: standard deviation
ANOVA: analysis of variance
chr: chromosome.
