# Supplementary material for: A novel circular RNA (hsa_circ_0059930)-mediated miRNA–mRNA axis in the lipopolysaccharide-induced acute lung injury model of MRC-5 cells
Source: Bioengineered. 2021 May 18;12(1):1739–51. doi: 10.1080/21655979.2021.1916276 (PMC8806270; doi:10.1080/21655979.2021.1916276)
Supplement: Supplemental Material [file KBIE_A_1916276_SM8302.zip › supplement/Highlights.docx]

**Highlights**

1. LPS-treated MRC-5 cells can be the cell model of ALI.
2. circ_0059930 was significantly upregulated in LPS-treated MRC-5 cells.
3. Silence of circ-0059930 promotes proliferation but inhibits apoptosis in ALI.
4. circ_0059930/miR-382-5p/TOP1 might be potential regulatory axis in ALI.
