# Supplementary material for: A novel circular RNA (hsa_circ_0059930)-mediated miRNA–mRNA axis in the lipopolysaccharide-induced acute lung injury model of MRC-5 cells
Source: Bioengineered. 2021 May 18;12(1):1739–51. doi: 10.1080/21655979.2021.1916276 (PMC8806270; doi:10.1080/21655979.2021.1916276)
Supplement: Supplemental Material [file KBIE_A_1916276_SM8302.zip › supplement/List of abbreviation.docx]

LPS: lipopolysaccharide

circRNA: Circular RNA

ALI: acute lung injury

GO: gene ontology

KEGG: kyoto encyclopedia of genes and genomes

CCK-8: cell counting kit-8

TOP1: topoisomerase 1

SIRS: systemic inflammatory response syndrome

ALI/ARDS: acute lung injury/acute respiratory distress syndrome

ncRNAs: non-coding RNAs

ceRNAs: competing endogenous RNAs

miRNAs: microRNAs

FBS: fetal bovine serum

NC: negative control

siNC: siRNA negative control

PBS: phosphate belanced solution

1. AAD: 7-Aminoactinomycin D

RIPA: radio-immunoprecipitation assay

BCA: bicinchoninic acid

SDS-PAGE: sodium dodecyl sulfate polyacrylamide gel electrophoresis

PVDF: polyvinylidene fluoride

ECL: electrochemiluminescence

DAVID: Database for Annotation, Visualization and Integrated Discovery

RT-PCR: reverse transcription polymerase chain reaction

qRT-PCR: quantitative RT-PCR

cDNA: complementary DNA

SD: standard deviation

ANOVA: analysis of variance

chr: chromosome
