# Supplementary figures and images for: A novel circular RNA (hsa_circ_0059930)-mediated miRNA–mRNA axis in the lipopolysaccharide-induced acute lung injury model of MRC-5 cells
Source: Bioengineered. 2021 May 18;12(1):1739–51. doi: 10.1080/21655979.2021.1916276 (PMC8806270; doi:10.1080/21655979.2021.1916276)

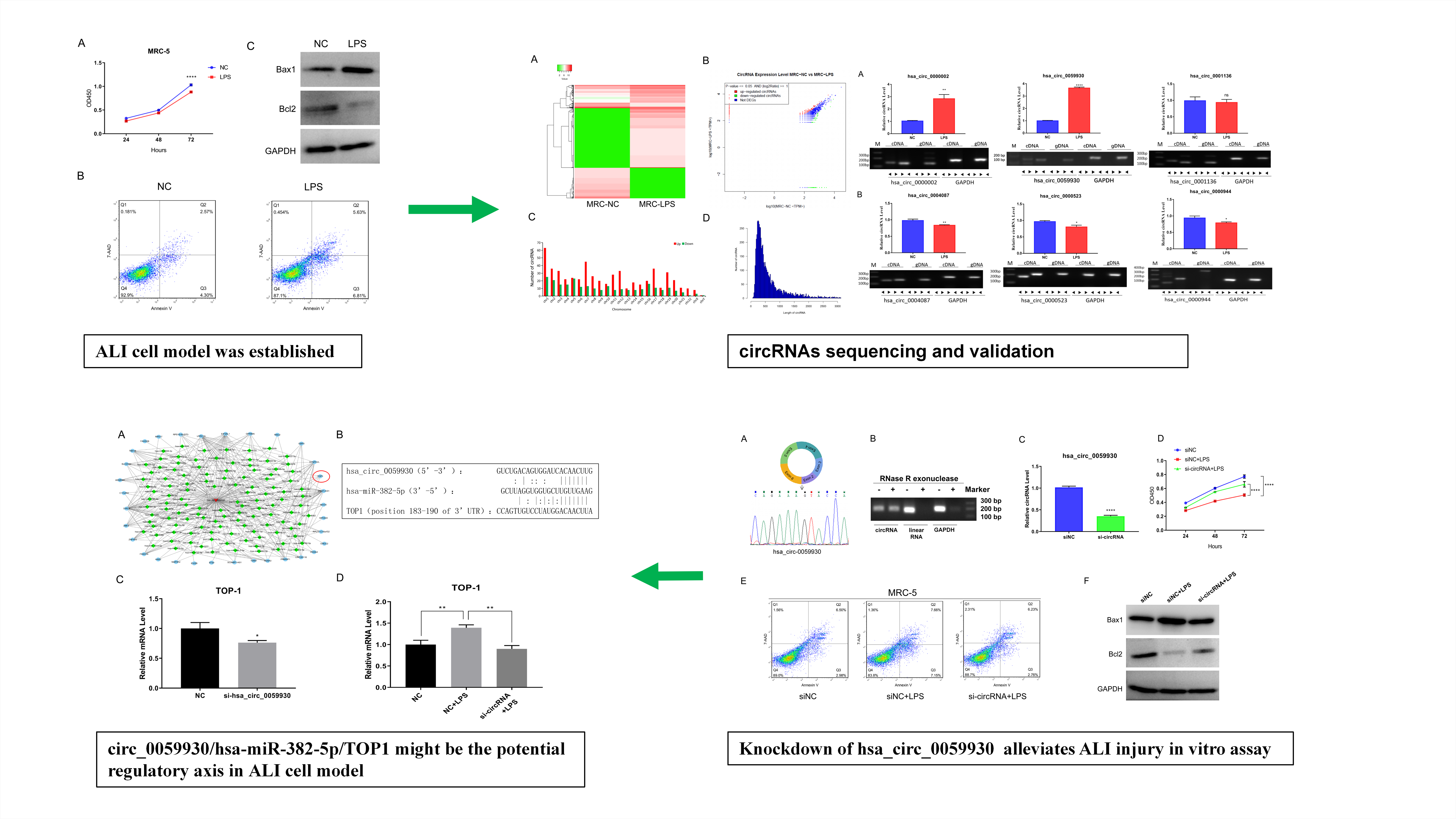

Supplement: Supplemental Material [file KBIE_A_1916276_SM8302.zip › supplement/Graphical Abstract (1).tif]
